# Slug Controls Stem/Progenitor Cell Growth Dynamics during Mammary Gland Morphogenesis

**DOI:** 10.1371/journal.pone.0053498

**Published:** 2012-12-27

**Authors:** Mayssa Nassour, Ysia Idoux-Gillet, Abdelkader Selmi, Christophe Côme, Maria-Luisa M. Faraldo, Marie-Ange Deugnier, Pierre Savagner

**Affiliations:** 1 Institut de Recherche en Cancérologie de Montpellier, Institut National de la Santé et de la Recherche Médicale U896, Université Montpellier, Centre Régional de Lutte contre le Cancer Val d’Aurelle-Paul Lamarque, Montpellier, France; 2 Institut Curie UMR144, Centre National de la Recherche Scientifique, Paris, France; Baylor College of Medicine, United States of America

## Abstract

**Background:**

Morphogenesis results from the coordination of distinct cell signaling pathways controlling migration, differentiation, apoptosis, and proliferation, along stem/progenitor cell dynamics. To decipher this puzzle, we focused on epithelial-mesenchymal transition (EMT) “master genes”. EMT has emerged as a unifying concept, involving cell-cell adhesion, migration and apoptotic pathways. EMT also appears to mingle with stemness. However, very little is known on the physiological role and relevance of EMT master-genes. We addressed this question during mammary morphogenesis. Recently, a link between Slug/Snai2 and stemness has been described in mammary epithelial cells, but EMT master genes actual localization, role and targets during mammary gland morphogenesis are not known and we focused on this basic question.

**Methodology/Principal Findings:**

Using a Slug–lacZ transgenic model and immunolocalization, we located Slug in a distinct subpopulation covering about 10–20% basal cap and duct cells, mostly cycling cells, coexpressed with basal markers P-cadherin, CK5 and CD49f. During puberty, Slug-deficient mammary epithelium exhibited a delayed development after transplantation, contained less cycling cells, and overexpressed CK8/18, ER, GATA3 and BMI1 genes, linked to luminal lineage. Other EMT master genes were overexpressed, suggesting compensation mechanisms. Gain/loss-of-function in vitro experiments confirmed Slug control of mammary epithelial cell luminal differentiation and proliferation. In addition, they showed that Slug enhances specifically clonal mammosphere emergence and growth, cell motility, and represses apoptosis. Strikingly, Slug-deprived mammary epithelial cells lost their potential to generate secondary clonal mammospheres.

**Conclusions/Significance:**

We conclude that Slug pathway controls the growth dynamics of a subpopulation of cycling progenitor basal cells during mammary morphogenesis. Overall, our data better define a key mechanism coordinating cell lineage dynamics and morphogenesis, and provide physiological relevance to broadening EMT pathways.

## Introduction

Epithelial-mesenchymal transition (EMT) is defined by a rapid change of cell phenotype. Epithelial cells typically loosen cell-cell adhesion structures, assume a motile pattern and elude apoptosis [1]. EMT has emerged as a unifying concept based on embryological studies. A set of genes, called “EMT master genes” has been characterized including transcription factor families Snail, Twist, Zeb and others [2]. However, in recent years, these genes have been found to be involved in distinct cell responses. Accordingly, EMT pathways appear to mingle with early differentiation pathways and stem cell maintenance or emergence [3], [4]. Here, we focused on transcription factor Slug (Snai2), that we characterized earlier [5]. The Snail family also includes Snail (Snai1) and Smuc (Snail3) genes [6]. Slug has been linked to early differentiation and morphogenesis in several cell types, including neural crest cells [7], [8], presomitic mesoderm [9] and atrioventricular canal endothelial cells, during heart morphogenesis [10]. In mammary epithelial cells, we found high levels of Slug in primary cells, contrasting with most transformed cell line models [11]. Accordingly, Slug has been found in the basal-like cell fraction obtained by CD24/CD49 or CD49/CD61 based-FACS analysis of mouse mammary epithelial cells [3]. Slug expression is also linked to an immature CD44 med/CD24 low phenotype in human mammary epithelial cells [12].

Slug is involved in cell motility in reepithelializing basal keratinocytes, pending Erk5 activation [13] and tumor progression, including mammary carcinoma [11], [14] and sarcoma [15]. Recently, Slug expression in breast carcinoma has been associated to a poorly differentiated phenotype in basal-like carcinoma [16], [17] and carcinosarcoma [18].

In this study, we focused on physiological roles for Slug during mammary gland morphogenesis. The mammary epithelium is organized as a bilayer, composed of basal/myoepithelial and luminal cells. During puberty, ductal morphogenesis results from the progression of a group of cohesive basal cells, the cap cells, located on the front of the terminal end buds (TEB). Basal cap cells exhibit self-renewal and regenerative capacity when transplanted in vivo, displaying properties of long-lived multipotent stem cells [19]. TEB cells lead the growing tubule through proliferation, migration and apoptosis coordination. Cap cells express P-cadherin (Pcad), proliferate and appear loosely connected [20]. Following early morphogenesis, adult mammary gland goes through cyclic changes in response to hormonal signaling. Changes in hormonal signaling during gestation and lactation lead to a dramatic gland remodeling with the development of alveolar secretory structures. Mammary stem cells have been suggested to play a key role during all these events. The existence of mammary stem cells is inferred from in vivo transplantation experiments and lineage-tracing experiments [21], [22]. In adult tissue, basal cells rather than luminal cells are able to regenerate entire mammary gland suggesting that the basal cell layer contains multipotent stem-like cells. Several genes, including BMI1, GATA3, ELF5 and ER, have been found to control critical steps along lineage specification [21].

In this report, we localized Slug in a proliferating basal compartment during mammary gland morphogenesis. We analyzed mammary gland development in Slug-deficient mice to uncover several defects. We tested Slug role in controlling mammary epithelial cell expansion in vitro using gain or loss of function experiments. All together, these results emphasize a role for Slug in the dynamics of stem/progenitor cells renewal and/or maintenance.

## Results

### Slug, but not Snail, is Mostly Expressed in the Basal Mammary Epithelial Cell Compartment

**Figure 1:**
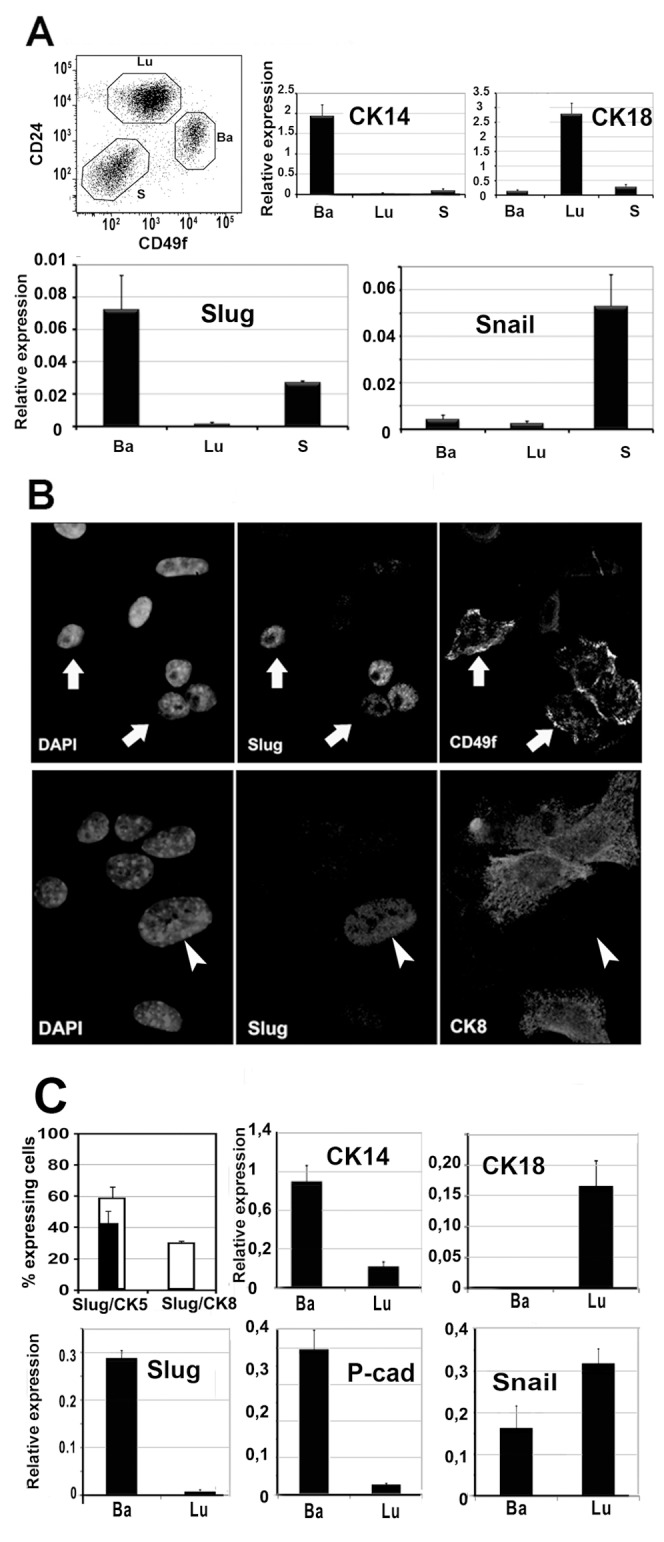
FACS and immunofluorescence analysis show Slug restricted expression by basal-like cells. A. Using FACS, primary mouse mammary cells were sorted into CD24 Neg (stromal cells: S), CD24 High/CD49f Med (luminal-like cells: Lu) and CD24 Med/CD49 High (basal-like cells: Ba). Results of two experiments are averaged to show expression levels of CK14, CK18, Slug and Snail as quantified by RT-qPCR and normalized to GAPDH reference gene. B. Double immunofluorescence study of mouse mammary epithelial cell line CommaDβ cells show co-expression of Slug and CD49f by a defined sub-population (arrows). In contrast, Slug and CK8 are found mutually exclusive from each other (arrowheads). C. Double immunofluorescence study was quantified for CK5 (white bar), CK8 (white bar) and co-expressed Slug (black bar inside white bar). No CK8+ cell were found to coexpress Slug. CommaDβ were also sorted based on Sca-1 expression as previously described (Deugnier et al., 2006). Sca-1-high basal-like (Ba) and Sca-1-low luminal-like (Lu) cell populations were analyzed for CK14, CK18, Slug, Pcad and Snail. Expression levels obtained by RT-qPCR were normalized to GAPDH reference gene. Data are shown as the mean (+sd) of 3 independent immunobead sorting experiments.

We performed FACS analysis to investigate simultaneously Snail, Slug and Smuc expression in freshly isolated mouse mammary epithelial cells using immunodetection of CD24 and CD49, two antigens known to discriminate basal/myoepithelial and luminal populations. As expected, the CD24 med/CD49 high fraction, called the basal fraction (Ba) and enriched in stem/progenitor cells, expressed cytokeratin (CK) 14 and not CK18 (Fig. 1A). Conversely, the CD24 high/CD49 low fraction, called luminal fraction (Lu), expressed CK18 but essentially no CK14. Slug was found to be only expressed by the basal fraction: CD24 med, CD49 high, CK14 high, CK18 neg. Snail was expressed at low and similar level in basal and luminal fractions, and mostly expressed in the stromal fraction (S). Smuc was only expressed at a very low level in the basal fraction (data not shown). In addition, we scrutinized Slug expression in an immortalized pluripotent mammary epithelial cell line called CommaDß. CommaDß cells can undergo functional differentiation in vitro and in vivo, including terminal end bud formation and casein synthesis. As we described before [23], these cells can be sorted into basal-like and luminal-like fractions using SCA-1 expression level. We first located Slug within the CK5 (basal marker), CD49f (basal marker) and CK8 (luminal marker) fractions by double immunofluorescence, using a validated anti-Slug antibody (Fig. S1). All Slug-expressing cells co-expressed CK5 and CD49f, none coexpressed CK8 (Fig. 1, B, C). Then, we sorted basal-like and luminal-like fractions using SCA-1 expression level. Similarly to primary cells, the basal-like fraction, identified by CK14 and Pcad expression, was found to include about all Slug-expressing cells (Fig. 1C). Snail was not found to be preferentially expressed by a cell fraction.

### Slug is Expressed by a Subpopulation of Basal Cells during Mammary Gland Morphogenesis

**Figure 2:**
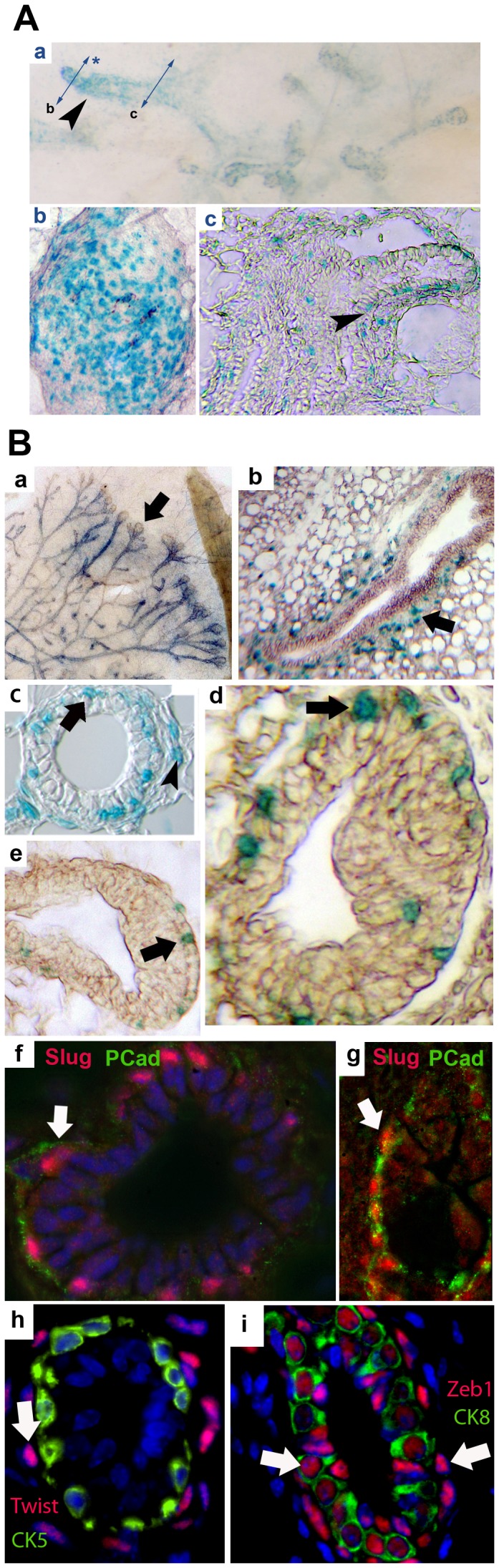
Slug, Twist and Zeb1 localization during mammary gland morphogenesis. A. At 3 weeks, Slug expression (blue X-Gal staining) is visible in the primary duct on a mammary gland wholemount (a, arrowhead), and section (b). Lower sectioning level (c) show Slug expression in polarizing basal epithelial cells during initial tubulogenesis (arrowhead). B. During mammary tubulogenesis at 6 weeks, Slug is found in growing tubules in wholemount (a) and tubule sections (b–c) involving epithelial cells (c, arrow) and peri-tubular mesenchymal cells (b, arrow and c, arrowhead). Cell location is better defined at higher magnification, including basal epithelial (arrow) and mesenchymal (arrowhead) cells (c). Tubule terminal end buds (TEB) sections also show expression by mostly basal epithelial cap cells (d–e, arrows). Co-labeling using DAPI (blue) and antibodies against Slug (red) and Pcad (P-cad, green) demonstrates a basal localization for Slug in 6 weeks (f) and adult (g) tubules. In addition, Twist (h) and Zeb1 (i) were located in tubules from 10 weeks-old mammary glands. Colabeling with CK5 show that only stromal cells express Twist (arrow). Conversely, CK8 colabeling show that Zeb1 is expressed by basal and luminal epithelial cells (arrows).

We located Slug at cellular level in growing mammary glands, combining two methods: ßGal activity in transgenic SlugLacZ mice [24] and immunolocalization. At early stages (3 weeks), Slug is expressed by numerous epithelial cells inside the primary duct as seen by wholemount observation and sectioning (Fig. 2A). Sectioning further down the primary duct (Fig. 2Ac) reveals Slug expression in polarizing epithelial cells during initial tubulogenesis (arrowhead). At 6 weeks, Slug is expressed in growing tubules in wholemount samples (Fig. 2Ba). Sections show this expression involves a large number of mesenchymal cells flanking the tubule (Fig. 2Bb, arrow; Fig. 2Bc, arrowhead). However, sections also show a strong staining by a significant subpopulation of basal duct cells (arrow Fig. 2Bc ) and cap cells (basal epithelial cells located at the tip of the growing TEB, arrow Fig. 2B, d–e). Overall, ßGal staining involved a sub-population estimated to 10–20% of basal cells. To avoid possible confusion with neighboring mesenchymal cells, we performed a triple labeling combining DAPI (blue) and antibodies against Slug (red) and P-cadherin (Pcad), specifically expressed by basal myoepithelial cells in the mammary gland (green on Fig. 2B, f–g). This co-staining demonstrates a basal localization for Slug expressing epithelial cells. To analyze a putative linkage with cell cycling and proliferation, we co-stained 7 weeks old mammary gland sections with anti-Slug and anti-PCNA1 antibodies. We found that 70% (+/−10%) of Slug-expressing cells were also expressing PCNA1 (Fig. S2A) when 73% (+/−5%) of PCNA1-expressing cells co-expressed Slug suggesting a positive link between slug expression and cell proliferation. Finally, we looked by immunofluorescence for the other EMT master gene families : Twist and Zeb. Twist was not observed in basal epithelial cells expressing CK5, but in peri-tubular fibroblast-like stromal cells, (Fig. 2Bh, arrow). Zeb1 was expressed by both basal and luminal epithelial cells, as confirmed by double labeling using anti-CK8 antibodies (Fig. 2Bi, arrows). Zeb2/SIP1 was described previously to not be expressed in breast [25]. All together, these results demonstrate Slug expression by a discrete sub-population of mostly proliferating mammary basal epithelial cells comprising about 10–20% of basal cells in vivo and characterized by the co-expression of CD49f, CK5 and predominantly PCNA1.

### Slug is Required for Normal Mammary Gland Tubulogenesis and Differentiation

**Figure 3:**
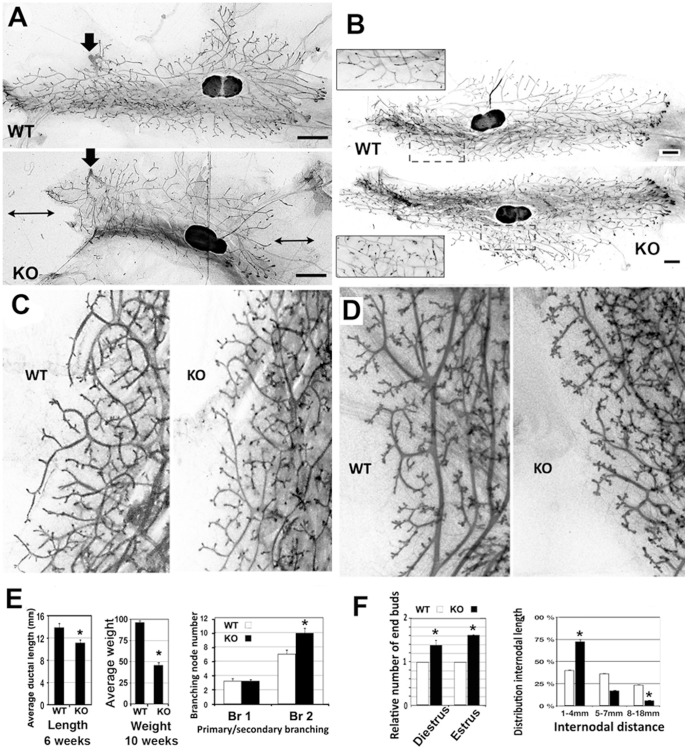
Mammary glands from Slug-deficient mice exhibit several defects. A. Mammary glands from wild type (WT) or SlugKO (KO) mice were analyzed in wholemount preparations during tubulogenesis (6 weeks old mice). KO mammary gland displayed a growth delay visible (double arrows) downstream and upstream from the primary duct (arrow). More than 6 mice were examined with a similar phenotype. Size bar = 1 mm. B. At later stages (8 weeks), wholemount preparations show a recovery linked to overbranching visible at higher magnification (inserts). C–D. Orthotopic grafts combining explants from wild type (WT) or SlugKO (KO) were examined 15 weeks after transplantation during estrus cycle (C), including diestrus phase (D). A significant increase (*Student test, p<0.05) in the terminal branching pattern is found in explants from KO mammary glands at all stages. E. Mammary gland phenotype was quantified by several ways. Average length of 12 primary ducts was estimated based on wholemount preparations from three 6 weeks old-mammary glands. In addition, 5 SlugKO and 9 WT (10 weeks old) mammary glands were weighed and compared. We also determined the number of branching nodes, discriminating between primary and secondary branching, significantly more abundant in KO explants (*Student test, p<0.05). F. Finally, we quantified in the explants the increase in the number of terminal buds in homozygous (black, KO) as compared to wild-type (white, WT) transplants, as determined in 5 KO explants from mammary glands at diestrus stage or estrus phases. Transplanted mammary glands from Slug-deficient mice also express extraneous branching. Internodal segments were sorted into short segments (1–4 mm), medium segments (5–7 mm) and longer segments (8–15 mm). Short segments, resulting from an increase in branching nodes generation, were clearly predominant in KO transplants (*Student test, p<0,05).

Homozygous SlugLacZ mice represent functional Knock-out (KO) [24]. We analyzed mammary gland morphogenesis at several developmental stages in homozygous SlugLacZ (SlugKO) mice. At 6 weeks, mammary epithelial terminal end buds (TEB) from normal or heterozygous mice had filled about two third of the mammary fat pad (Fig. 3A). However, SlugKO mice tubulogenesis was delayed significantly (Fig. 3A, double arrows). Based on 6 SlugKO mice, primary ducts were significantly shorter by 20% (Fig. 3E). Nevertheless, a smaller mammary fat pad was filled by 10 weeks in all mice. Recovery was accompanied by a light but distinct overbranching pattern in SlugKO mice (Fig. 3B). Overall, SlugKO mammary glands remained significantly smaller and lighter at the end of tubulogenesis at 10 weeks. Average weight was decreased by about half (Fig. 3E) in a cohort of 14 mice. Tubules from SlugKO mice showed the standard histological organization with basal and luminal cell layers, according to smooth muscle actin (SMA) and CK5 (basal markers) or CK8 (luminal marker) expression pattern (data not shown).

We investigated further the mammary phenotype using classic transplantation experiments [26]. We grafted epithelial fragments derived from 12 weeks old SlugKO or control wild type (WT) mice into mammary fat pads from 9 WT 3-weeks-old mice previously depleted from the epithelial rudiment. In each case, paired epithelial fragments from SlugKO and WT mice were transplanted on each side, contralaterally, into inguinal mammary fat pads from a recipient mice to be compared with genuine mammary gland from the recipient mice, exposed to the same hormonal environment. After 6 weeks, both grafted glands had grown to a comparable extent. The growth delay observed in homozygous mice was not seen in grafts suggesting that Slug-deficient mice may display systemic and/or mammary stromal defects. The limited overbranching phenotype was present in grafted SlugKO explants (Fig. 3C–F). Two of the five grafted mice examined after 10 weeks were in diestrus and displayed the expected increased branching. It was significantly denser in homozygous-derived explants (Fig. 3D). We quantified the overbranching by several ways detailed in Fig. 3E–F, emphasizing a distinct but limited phenomenon. We then examined physiological abilities of Slug-deficient mammary glands. We found that grafted mammary glands from both WT and SlugKO mice were able to some extent to respond to physiological signaling during pregnancy and produce milk in alveoli (data not shown). Taken together, these results show a drastic 50% decrease in mammary gland size, matched by a distinct but modest overbranching. They suggest a morphogenetic role for Slug during mammary gland tubulogenesis and differentiation.

### Slug Regulates Genes Involved in Mammary Epithelial Cell Lineage

**Figure 4:**
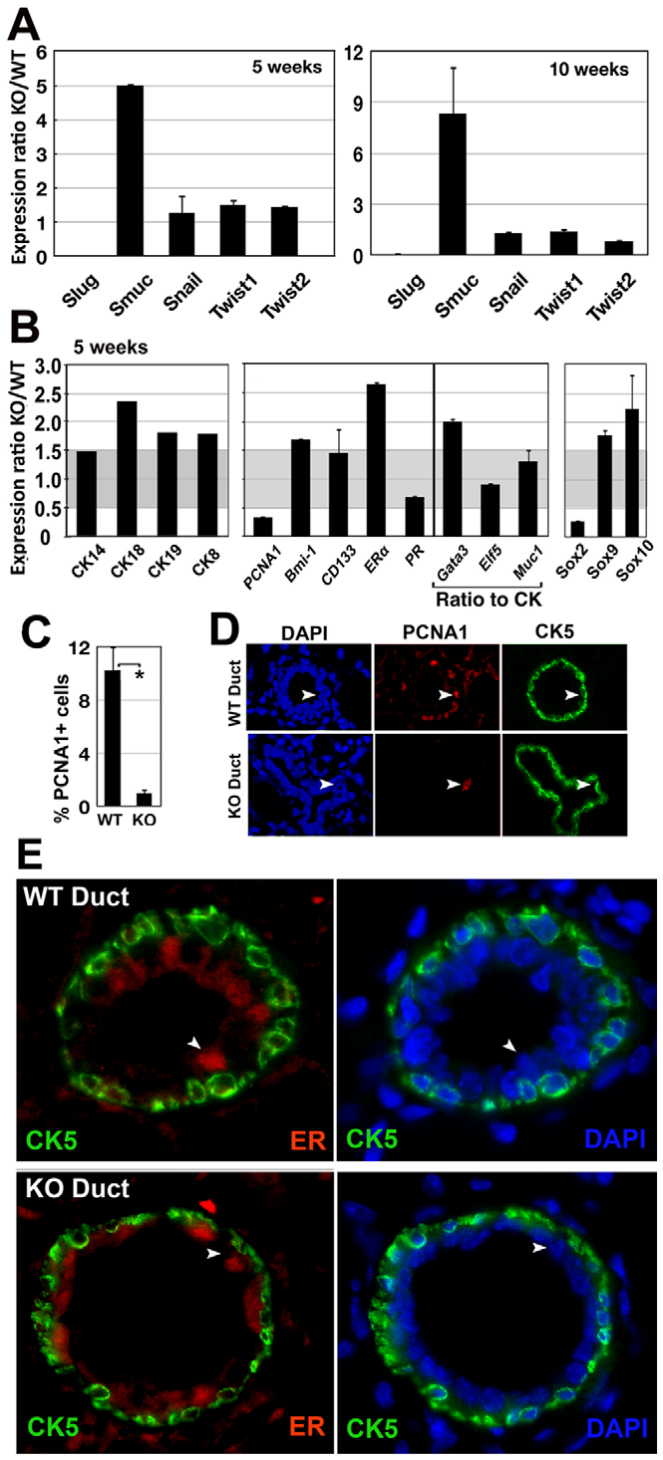
Slug regulates mammary epithelial cell differentiation pathways. We quantified expression levels of various genes involved in mammary epithelial cell differentiation in WT and KO mammary glands from same litter mice. Two KO mice from the same litter were used for the 5 weeks study and the expression level averaged. A. EMT-master genes from Snail and Twist families were screened at both stages. B. Basal (CK14) and luminal (CK 8, 18, 19) cytokeratin genes were screened to evaluate the relative epithelial fraction and putative regulation at 5 weeks. Genes reflecting proliferation (PCNA1), stem/progenitor or luminal phenotype (Bmi1, CD133, Gata 3, Elf5, Muc1, ER, PR) were also screened at 5 weeks. Because CK average expression level was significantly increased, we related the expression levels from strictly epithelial genes (Gata3, Elf5, Muc1) to the mean CK expression level. Finally, members of the Sox family of transcription factors were also screened. Gray areas cover gene expression ratios considered as not significantly modified (ratio between 0.5 and 1.5). C. PCNA1 expression was quantified by calculating the percentage of cells expressing PCNA1 among 1–500 cells in growing ducts paraffin sections from 5–8 weeks old mice, showing a strong decrease in SlugKO cells (*Student test, p<0,05). D. PCNA1 and CK5 were visualized by double-immunofluorescence including DAPI co-staining on paraffin sections from wild-type (WT) or Slug-deficient mouse (KO) ducts, as indicated. E. ER was colocalized with CK5 and DAPI to show a relative overexpression in Slug-deficient mouse (KO) ducts. Arrows points to ER+ CK5- cells identified as luminal cells.

We collected mammary gland RNA from same litter WT or SlugKO mice and used RT-qPCR analysis to scrutinize genes involved in mammary morphogenesis. We first analyzed 5 and 10 weeks-old mammary glands to scrutinize transcription factors linked to the snail family. We added two functionally related members of the Twist family. Slug was totally suppressed, as expected. Smuc, was increased 5 to 8 times in KO mice (Fig. 4A). In addition, Twist genes were found to be upregulated in a few mice mammary glands when Smuc was not modulated (data not shown), emphasizing individual heterogeneity. We then focused on 5 weeks old mammary glands to avoid potential interference with hormonal cycle. We scrutinized cytokeratins to find upregulation of luminal cytokeratins CK18, 19, 8 in KO mice (Fig. 4B). To take into account the relative increase in the epithelial compartment, strictly epithelial genes including Gata3, Elf5 and Muc1 were reported to the CK mean expression level, (all CK combined). We found that BMI1 and GATA3, predominantly implicated during luminal epithelial cell differentiation were upregulated in KO mice. We also found that PCNA1, a marker of cell proliferation, was significantly downregulated. The link between Slug expression and proliferation rate was examined further by counting PCNA1-expressing cells in tubules sections from mutant mice. Using a Student test, we found significantly less PCNA1+ cells in SlugKO ducts (Fig. 4C–D) at 5 and 8 weeks. Similar downregulation was observed with KI67 and at later stages (data not shown). Considering the smaller size and weight of SlugKO mammary glands, this decrease remained compatible with observed tubulogenesis.

We also monitored Sox genes involved in stem/progenitor cell dynamics to uncover a down-regulation of Sox2 in KO mice when Sox9 and Sox10 were upregulated (Fig. 4B). We checked that Sox 9, similarly to GATA3 and Muc1 was strictly expressed by epithelial cells. Colabeling with anti-CK5 or anti-CK8 antibodies confirmed that only luminal cells expressed GATA3 and Muc1, when Sox 9 was found in basal and luminal cells, more abundant in luminal cells in terminal buds (Fig. S2).

Estrogen receptor was also upregulated and this overexpression was confirmed by immunofluorescence study (Fig. 4E). Only luminal cells (negative for CK5, arrowhead) were found to express ER.

Those observations imply a role for Slug in controlling the dynamics of a subpopulation of basal progenitor cycling cells, apparently restricting luminal differentiation. They also evoke compensation mechanisms in SlugKO mice, resulting in individual heterogeneity.

### Slug Controls Clonal Mammosphere Growth

**Figure 5:**
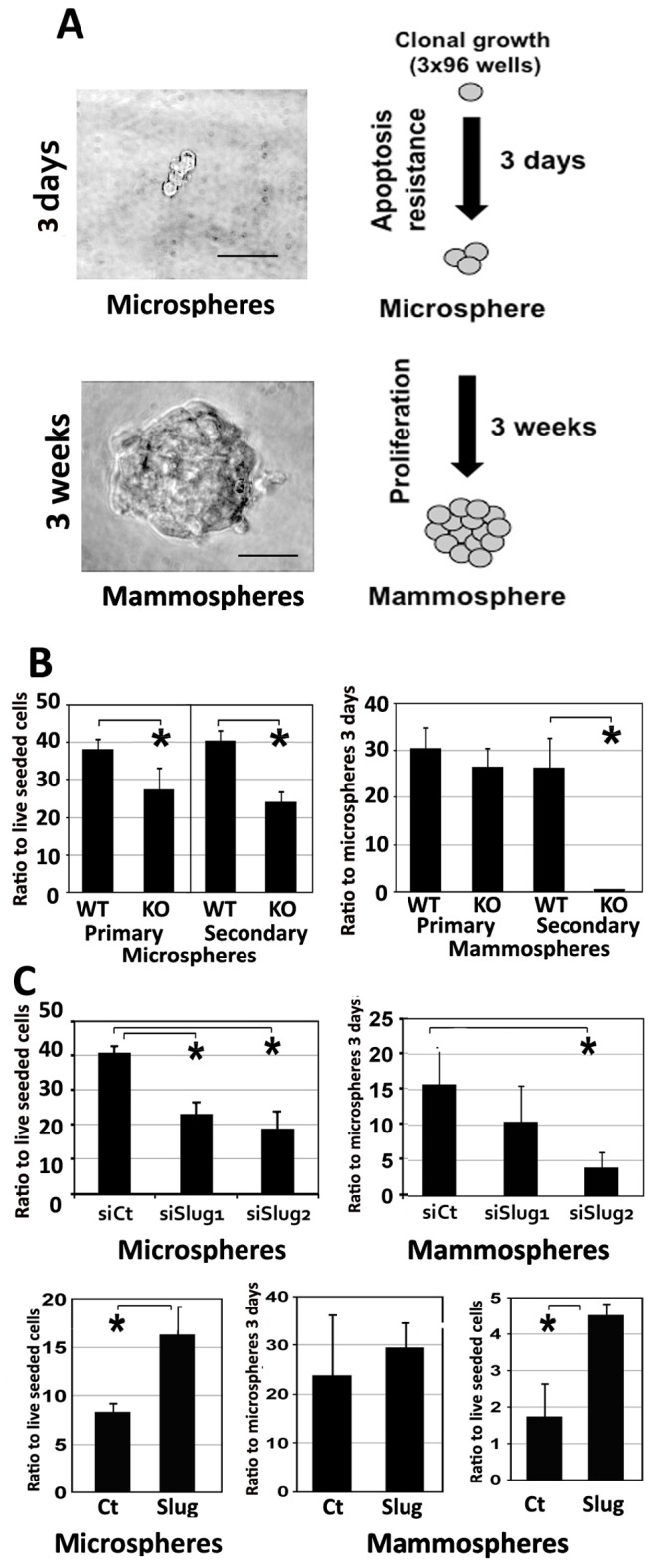
Slug controls mammosphere growth. A. Epithelial cells isolated from mammary glands were seeded individually in low-adherence culture wells. All wells were screened after 3 days. Wells containing 1–4 live cells (microsphere) were enumerated. After three weeks, Some of the microspheres evolved into mammospheres that were monitored. Values are reported as percentage compared to the number of live seeded cells or to the number of microspheres at 3 days. Bar = 50 µm. B. Cells from wild-type (WT) or Slug-deficient mouse (KO) were cloned to grow into primary microspheres and mammospheres. For secondary mammospheres, 10–20 primary mammospheres were pooled and enzymatically dissociated before individual cloning and further screening. Experiment was repeated three times. C. CommaDβ cells were transfected with control (siCt) or two distinct anti-Slug siRNA (siSlug1 and siSlug2) and seeded individually in 96 well low-adherence dishes. All wells were screened after 3 days. Microspheres and mammospheres were reported separately after 3 weeks. A significant decrease in viability was observed after 3 days in cells transfected with anti-Slug siRNA. This deficit was even stronger three weeks later (*Student test, p<0.05). CommaDβ cells were also transfected with control (Ct) or Slug full-length cDNA (Slug) expression vectors and seeded individually in 96 well dishes as previously. The number of mammospheres showed a significant and early increase in survival and growth for Slug-overexpressing mammospheres (*Student test, p<0.05).

One characteristic associated with mammary stem/progenitor cells is their ability to grow clonally in suspension and establish so-called mammospheres [27]. We examined Slug suppression effect on mammosphere growth and survival in stringent conditions (Fig. 5). Primary mammary epithelial cells were isolated and grown under clonal conditions (2 dissociated cells per well). All wells were blindly checked after three days to identify wells containing live cells (one to four cohesive cells). These small clonal aggregates were called microspheres and monitored to evaluate early cell survival in clonal conditions. Cells were then left to grow for 3 weeks, then blindly screened to enumerate mammospheres (>50 µm) that grew out from the microspheres (Fig. 5A). In addition, in order to generate secondary/tertiary microspheres and/or mammospheres, we dissociated some primary/secondary mammospheres. Isolated cells were then recloned in suspension in the same conditions. Overall, we found significantly less microspheres after 3 days in primary or secondary plating of KO mice mammary epithelial cells. However, the proportion of mammosphere growing after 3 weeks (related to the number of microsphere) was not significantly different starting from primary WT or KO mice mammary epithelial cells. This underscores the importance of cell survival during the initial seeding period. In contrast, no mammosphere were found to grow in secondary plating in 3 distinct experiments, when the percentage of secondary mammospheres starting from WT mammospheres was similar to the percentage of primary mammospheres (Fig. 5B). This drastic result emphasizes a gate keeper role for Slug in stem/progenitor cell renewal process in these conditions. We checked mammosphere composition by immunofluorescence and found that a majority was expressing cytokeratins. These studies also supported the clonal nature of the mammospheres since none were found to co-express cytokeratins and vimentin (Fig. S3).

In order to analyze Slug impact on mammosphere growth, we also suppressed or increased Slug expression in commaDβ cells (Fig. 5C). Two distinct sequences were selected inducing a significant Slug down-regulation at protein level. CommaDβ cells were transiently transfected with control (siCt) or two distinct anti-Slug siRNA (siSlug1 and siSlug2), then enzymatically dissociated and seeded individually into 3 low-adherence 96 multiwell dishes for each condition. As previously, microspheres were blindly reported after 3 days, and mammospheres after 3 weeks. Two to three times less microspheres and mammospheres were obtained when cells were transfected with anti-Slug siRNA sequences (Fig. 5C), a significant difference as found by a Student test (p<0.05). We also transiently overexpressed Slug in commaDβ cells, resulting in a 3–4 times increase in slug protein amount, as estimated by three western blot analysis (Fig. S1 and data not shown) and blindly examined clonal mammosphere growth as before in three distinct 96 wells dish for each conditions. We found that Slug overexpression induced about twice more microspheres after 3 days, resulting in about 2–3 times more mammospheres (Fig. 5C). This significant increase (p<0.05, Student test) reflected the early microsphere raise, since the ratio to 3 days-microspheres was not significantly different. We also quantified the increase in microsphere/mammosphere growth after secondary or tertiary cloning. A significant increase in the number of mammospheres was observed in link with the number of passages (data not shown). This confirms, as noticed in other reports [27], that mammosphere growth conditions favors emergence of self-seeding cells. All together, these results suggest Slug controls mammosphere growth, mostly by promoting early cell survival in suspension conditions, a gate-keeper role in these conditions. It also suggests that Slug plays a critical role in stem/progenitor-like cell self-renewal, a requirement for secondary mammosphere growth.

### Slug Regulates Mammary Cell Proliferation, Apoptosis, Differentiation and Migratory Potential

**Figure 6:**
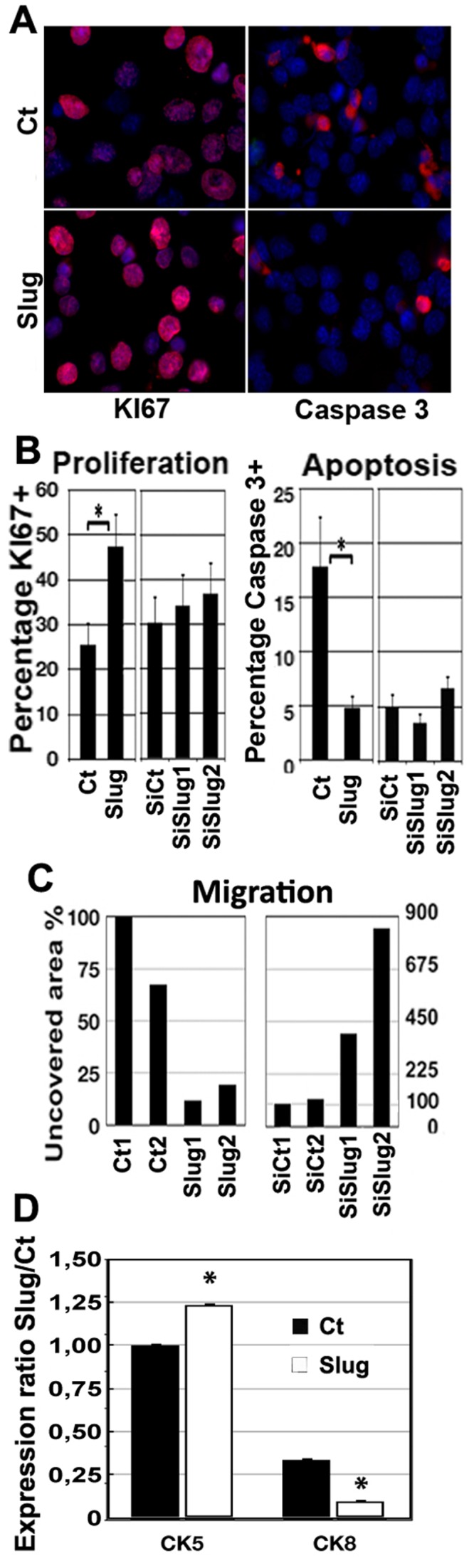
Slug controls cell proliferation, apoptosis, motility and cell lineage commitment. A-B. CommaDβ cells were transfected with control (Ct) and Slug expression vector (Slug), or with si control (siCt) and two distinct anti-Slug siRNA (siSlug1 and siSlug2). After fixation, DAPI staining and immulocalization (A), average percentage of cells expressing KI67 or caspase 3 were calculated and reported (B). C. Slug controls mammary epithelial cell motility. Cell motility was estimated using a wound healing assay in confluent CommaDβ cells. Ability to repopulate the wound area was estimated by measuring total uncovered substrate area after 48 h, reported to 0 h. Cells were transfected as indicated with Slug full-length cDNA (duplicate Slug1 and Slug2), control vector (duplicate Ct1 and Ct2), and with anti-Slug siRNA (siSlug1 and siSlug2), and two distinct controls siCt1 and siCt2. Experiment was repeated three times. D. Slug controls mammary epithelial cell commitment. Basal/Luminal differentiation phenotype was evaluated by immunofluorescence using cytokeratin expression pattern. CK5 (basal) was found to be significantly overexpressed in cells overexpressing Slug (Slug) as compared to control (Ct), when CK8 (luminal) was downregulated in cells overexpressing Slug.

Mammosphere growth results from survival and proliferation potential in selective conditions. In order to analyze Slug control over mammosphere growth and morphogenesis, we scrutinized primary mammary epithelial cells and CommaDβ cells, treated to raise or lower Slug expression level. We evaluated proliferation and apoptosis by calculating cell percentage expressing KI67 or caspase 3. We found no significant difference in plated primary cells derived from WT or KO mice (data not shown). Conversely, Slug-overexpressing CommaDβ cells displayed significantly more KI67+ cells and less caspase 3+ cells, suggesting more cycling and less apoptotic cells (Fig. 6A–B). This enhancing effect on cell proliferation reenforces our in vivo observation. Slug inhibition by siRNA interference had no significant effect in these conditions. To evaluate a possible modulation of the basal/luminal lineage, we checked the expression levels of cytokeratins in slug-overexpressing CommaDβ cells. We found that luminal cytokeratin CK8 was significantly down-regulated when basal cytokeratin CK5 was upregulated (Fig. 6D). This suggest that Slug overexpression favored a basal phenotype determination.

Recognizing that tubulogenesis also involves cell motility, we scrutinized Slug influence on cell migratory potential by performing wound healing assays. Confluent CommaDβ cells were wounded, then transfected with antiSlug siRNA sequences. Migration was defective in Slug-silenced cells, as quantified by measuring uncovered wound area in the culture dish (Fig. 6C and Fig. S4). Since proliferation is not significantly affected in Slug-silenced cells, we can postulate the effect on cell migration is about cell motility. We also overexpressed Slug in CommaDβ cells, previous to testing their migrating ability. We found that Slug overexpression was linked to a faster and more extensive migration (Fig. 6C and Fig. S4). In summary, Slug appears to support cell proliferation and migration, and to repress apoptosis in pluripotent mammary epithelial cells, in accordance with our in vivo observations.

## Discussion

In this report, we uncover a new mechanism linking EMT to stem/progenitor cell dynamics in mammary gland. This pathway involves Slug, an EMT master gene, member of the Snail family. We localized Slug in vivo in mammary glands at cellular level, using both immunolabeling and a transgenic mouse. We found Slug within a subpopulation of about 10–20% basal epithelial cells (Fig. 2B). Such a value appears to be above the estimated number of “true” stem cells in the mammary gland (1000–14,000) [21], suggesting Slug expression is not restricted to this population, but may include it. The high number of Slug-expressing cells in the primitive duct at early stages (Fig. 2A) suggests a wider expression pattern linked to morphogenetic phases preluding to further epithelial differentiation in accordance with Slug expression during somite emergence [9] and cutaneous reepithelialization [28]. We observed in vivo that Slug was mostly expressed in PCNA1+ cells and that PCNA1 was downregulated in SlugKO mammary glands during tubulogenesis, suggesting that Slug is associated in vivo with a proliferative territory (Fig. 2B).

The impact of Slug on epithelial differentiation was confirmed by analyzing primary and orthotopic grafts of SlugKO epithelial buds, resulting in smaller size, light overbranching and precocious differentiation (Fig. 3). These results support a direct involvement of Slug in progenitor cell dynamics, resulting in cell fate resetting, as suggested by slug overexpression experiments in a recent publication [29]. The relatively modest phenotype could reflect compensation mechanisms, suggested by the upregulation of other EMT master genes, SMUC and/or Twist in SlugKO mice (Fig. 4). EMT genes are known to be redundant and regulate each other [30]. Accordingly, we found E-cadherin, a typical molecular target for EMT master genes [31], to not be significantly modulated in SlugKO mice (data not shown).

Mammosphere growth experiments show a decreased clonogenic ability in SlugKO primary mammary epithelial cells grown in suspension, a property associated with stemness. In fact, we could not get secondary mammosphere to grow out of SlugKO primary mammospheres, suggesting stem cell self-renewal process was severely impacted in these stringent conditions (Fig. 5). Analogous conclusion was reached in Guo et al. (2012), using mammary cells stimulated by a matrigel-based substrate [29]. Clonal mammosphere growth, as analyzed in our defined and stringent conditions, implies both cell survival and proliferation. In accordance, less Slug-deficient cells survived after 3 days to give rise to microspheres, and less microspheres grew to become mammospheres after 3 weeks suspension culture. Reciprocally, we also found that Slug mild overexpression in mammary epithelial cells increased mammosphere growth and cell proliferation, and inhibited apoptosis after 2–3 days in 2D conditions (Fig. 6). These distinct mechanisms contribute to clonal mammosphere growth, accentuating a role as a gate-keeper for Slug in stem/progenitor cell expansion. Further work in vivo will test this hypothesis, but will have to take into account the plurifactorial compensation mechanisms suspected to take place in SlugKO mice.

We identified several targets for Slug in the mammary gland microenvironment (Fig. 4). We found that genes directly involved in epithelial stem cell dynamics and luminal differentiation, including Bmi1 [32], GATA3 [33] and cytokeratins CK8/18/19 are upregulated in SlugKO mice. Estrogen receptor was upregulated at tubulogenesis stages in SlugKO mice, in accordance with previous findings describing a negative feedback between ER and Slug pathways [34]. We found an intriguing decrease of Sox 2, associated with stemness, when Sox 9 was upregulated. Despite the recently described cooperation between Slug and Sox 9 in mammary gland [29], we found that cell territories expressing these factors were very distinct in mammary tubules. Sox10 upregulation in SlugKO mice is noticeable considering the basal-like distribution previously reported [35] and the putative role in mammary cell fating [36].

The link between Slug and maintenance/emergence of early differentiation stages is inferred by several published observations: Slug is overexpressed in embryo stem cells [37]. Slug is necessary for hemopoietic stem cells to survive and expand [38], [39]. Slug controls presomitic mesoderm differentiation into somite wall epithelial cells [9]. Slug is necessary for the amplification of basal keratinocytes during wound healing [28]. This role was confirmed by overexpression experiments in mammary epithelial cells, resulting in increased mammary tubule growth [29]. In human cancer, Slug is also linked to a basal phenotype. Slug overexpression modifies expression of CD44 in transformed epithelial cells grown in suspension [12]. Slug has been shown to be overexpressed in basal-like breast carcinoma [17], [40] involving NF-KappaB pathway. It has been proposed to play a role in controlling cancer cell phenotype commitment in a BRCA-mutant environment during basal-like breast carcinoma progression [40].

In conclusion, we characterized a novel role for the transcription factor Slug during mammary gland morphogenesis. Slug pathway appears to play a gate-keeper role and control the maintenance and dynamics of an amplifying stem/progenitor cell compartment during morphogenesis.

## Materials and Methods

### Mice

The Slug-LacZ mouse line was initially generated by inframe insertion of the β -galactosidase gene into the zinc finger coding region of the Slug gene [24] and graciously provided by Dr. Thomas Gridley (The Jackson Laboratory, Bar Harbor, ME).

### Ethics Statement

All animal procedures were conducted in strict adherence of European Convention STE 123 for the Protection of Vertebrate Animals Used for Experimentation (completed by ordinance n° 2001-486, 86/609/CEE, n° 87–848, and n° 2001-464) and approved by the «Comite d’Ethique de l’IRCM (INSERM)», which is the local ethics committee.

### Cell Culture

The Comma-D cell line was established from the normal mammary gland of a mouse at midpregnancy [41]. CommaDβ (passage 8) were derived from the parental Comma-D cells and graciously provided by Dr. Medina (Baylor college of Medecine, Houston, TX). They were routinely grown in DME F12 (Gibco> BRL) supplemented with 2% FCS (Gibco BRL), 2 mM L-glutamine (Gibco BRL), 10 µg/ml bovine insulin (Sigma), and 5 ng/ml murine EGF (Sigma). Cells were routinely subcultured at split ratios of 1∶5.

### Mammary Gland Wholemount

Mammary glands were harvested and spread on glass slides (SuperFrost Plus/Thermo SCIENTIFIC). Tissue was fixed with paraformaldehyde 4% in PBS for two hours at room temperature. Mammary glands were then stained overnignt in carmine alum solution, washed in EtOH 70%, EtOH 95% and EtOH 100% for 15′. Mammary glands were cleared in xylene overnight. Pictures were analyzed using Image J software (NIH).

### X-Gal Staining

For whole-mount X-gal staining, Slug–lacZ heterozygous and WT mice were sacrificed humanely by CO2 inhalation. Mammary glands were harvested and fixed with 4% paraformaldehyde in PBS, for 1 h at room temperature, washed 3 times (PBS 1X, MgCl2 0,2 mM, sodium-deoxycholate (DOC) 1X and NP-40 0,02%) and incubated at 30°C overnight with X-gal staining solution (1,5 mg/ml X-gal (Q-BIOgene), 10 mM K3Fe(CN)6 (sigma), 10 mM K4Fe(CN)6 (Sigma) diluted in the washing solution. Mammary glands were then post-fixed with 4% formaldehyde, dehydrated and were processed for paraffin inclusion. Sections of 5 µm were deparaffinized, and mounted with Mowiol 40–88 (Sigma).

### Transplantation of Mammary Epithelium

Cleared mammary fat pads were prepared in 3-week-old female nude Balb/c mice (Charles Rivers) by surgically removing the developing mammary epithelium of the fourth inguinal glands, as previously described [42]. Pieces of approximately 1 mm^3^ were dissected from the mammary fat pad region adjacent to the primary duct and containing mammary rudiment clearly visible with the dissection microscope (donor epithelium). These pieces were implanted into the inguinal number four fat pads. Pieces of tissue removed from the recipient fat pad were stained with Carmine to control for the elimination of endogenous epithelium. In each case, mutant (piece from Slug KO mouse) and control (WT) epithelia were grafted into the contralateral fat pads of the same recipient mouse. The outgrowths produced were analyzed by dissecting recipient fat pads 7–15 weeks after transplantation.

### Immunofluorescence

Mouse mammary gland samples were frozen in OCT medium (Tissue-Tek/SAKURA). Frozen sections of 5 µm were fixed in paraformaldehyde 4% for one hour and washed in PBS before antibody incubation. For paraffin embedded tissues, samples were fixed in paraformaldehyde 4% for 2 hours at room temperature before paraffin inclusion. Sections of 5 µm were deparaffinized, and were processed for antigen retrieval by incubation in citrate buffer (PH 6) at 95°C for 45 min. Slides were then washed in PBS before antibody incubation.

Cells were grown on glass coverslips for 48 h before fixation with methanol at −20°C for 6′ for CK immunolabeling. For Slug, Pcad, SMA, CD49f, PCNA1 immunostaining, cells were fixed and permeabilized for 5′ with paraformaldehyde 4%+triton (0.05%) for 5′ followed by further paraformaldehyde treatment (4%) for an additional 15′. After washing in PBS, cells or tissue sections were incubated for 1 hour at room temperature with primary antibodies, as indicated on Table S1. Several commercial anti-Slug antibodies were found to lack specificity and a preliminary careful validation was found essential (Fig. S1). Secondary antibody was added to cells for a 30′ incubation at room temperature. Non-specific staining was blocked by adding 10% goat serum to both primary and secondary antibodies. Examination was performed using a Leica DM IRB inverted microscope (Leica Microsystems) and images were acquired with a CoolSNAP HQ camera (Roper). Image acquisition and analysis involved a dedicated regional imaging platform, the Montpellier RIO Imaging (MRI).

### Preparation of the Mammary Epithelial Cells and Flow Cytometry

Mammary fat pads were mechanically dissociated with scissors and digested for 90 min at 37°C in CO_2_-independent medium (Invitrogen) supplemented with 5% fetal bovine serum, 3 mg/ml collagenase (Roche Diagnostics) and 100 U/ml hyaluronidase (Sigma). The resultant suspension was sequentially resuspended in 0.25% trypsin–EDTA for 1 min, and then 5 min in 5 mg/ml dispase (Roche Diagnostics) with 0.1 mg ml–1 DNase I (Sigma) followed by filtration trough a 40 µm mesh. Red blood cells were lysed in NH_4_Cl. To separate basal and luminal cells, mammary epithelial cells isolated from the inguinal glands of five 12-week-old virgin Balb/c mice were pooled, stained with anti-CD24-PE, anti-CD49f-FITC anti-CD45-APC and anti-CD31-APC antibodies, as described elsewhere (Mani et al. 2008). CD24-low/CD49-high (basal) and CD24-high/CD49-low (luminal) cells were purified using FACSvantage (Becton Dickinson) and used to isolate RNA to quantitatively evaluate gene expression, using the Montpellier RIO imaging facility. CD45- and CD31-positive stromal cells were also isolated from the flow cytometry analysis. Conjugated isotype-matching IgGs were used as negative controls.

### RNA Extractions and Reverse Transcription

RNA from cell lines were extracted using TRIzol® RNA reagent (Invitrogen). RNA quality was checked by spectrometer analysis and gel migration. One microgram of total RNA was then reverse transcribed using hexanucleotides in combination with Superscript II Reverse Transcriptase (Invitrogen).

### Real Time-Quantitative PCR

RT-qPCR was performed using an ABIPRISM 7300 (Applied Biosystems). Primer sequences are reported in Table S2. Amplifications were done using the SyberGreen master mix (Applied Biosystems) in 25 µl using standard PCR conditions (40 cycles with an annealing/elongation step at 60°c). Results are derived from at least two independent experiments or RT-qPCR estimations. Primary data generated by RT-qPCR were expressed as the difference in the number of cycles between the studied gene and a control housekeeping gene, 18 s, during the linear amplification phase. In some cases, values were related to a specific sample.

### Mammosphere Culture

The CommaDβ cells were trypsinized, and single cells were plated in ultralow attachment plates (Corning/Sigma) at a density of 2 cells/well in a serum free DMEM-F12 medium supplemented with B27 (Invitrogen), 20 ng/mL EGF (Fisher bioreagents), 4 mg/ml heparin (Fisher bioreagents) and 20 ng/mL bFGF (R&D). Cultures were fed every 4–5 days and mammospheres were counted after three weeks, defined as aggregates with a diameter >50 µm. In some cases, mammospheres were prepared by cytocentrifugation and analyzed for immunofluorescence after methanol fixation. Secondary mammospheres were prepared by dissociating about 10–20 mammospheres with trypsin/EDTA (0.05%) and seeding at clonal conditions (2 cells/well).

### Transfections

Plasmids: For transient transfection experiments, CommaDβ cells were tranfected by using the Lipofectamine 2000 protocol (Invitrogen). After incubation at 37°C/5% CO2 for 36–72 hours, Cells were harvested for preparation of total RNA or for protein extraction. The pEGFP-N2 control vector was used to determine transfection efficiency. M PCR3 Slug vector was used for Slug overexpression experiments [5].

SiRNA**:** Transfections were carried out by using INTERFERin (Polyplus) and 10 nM duplex siRNA per well, as per the manufacturer’s instructions. The siRNAs were synthesized by Eurogentec (sequences in Table S2). Two sequences of siRNA oligoribonucleotides were used for Slug (siSlug1 and siSlug2). The nonspecific siRNA oligoribonucleotides (siCt1, siCt2) were randomly synthesized and did not correspond to any known gene in the mouse genome database. After 48 h incubation, cells were collected for RNA and protein extraction.

### Western Analysis

The cells were lysed in a buffer containing Tris 10 mM, SDS 1%, EDTA 5 mM, β-glycerophosphate 10 mM, sodium pyrophosphate 10 mM and a protease inhibitors cocktail (Complete- Roche) or in ProteoJET™ Mammalian Cell Lysis Reagent with ProteoBlock™ Protease Inhibitor Cocktail (Fermentas). Cell debris were sedimented (13,000 g, 5 min). The protein content of the soluble fractions was determined with the BCA Protein assay Reagent kit (Pierce). Protein extracts (40 µg) were resolved by 12% SDS-PAGE (sodium dodecyl sulfate- polyacrylamide) gel as appropriate, and transferred to a nitrocellulose membrane (Amersham). Membranes were incubated with 5% nonfat dry milk or with 5% BSA for 1 hour at room temperature and probed with primary antibodies at 4°C overnight. Immune complexes were detected by chemiluminescence with HRP-conjugated secondary antibodies.

### In vitro Wound Healing Assay

Cells were grown to confluency in 24 well plates, transfected with siRNA or plasmids when necessary. Denudation zones were created by pushing the narrow end of a P200 plastic pipette tip through the cell monolayer. After wound healing, cells were fixed in paraformaldehyde 4% and colored with eosin and hematoxyline. Reepithelialization area was quantified by the difference between uncovered wound area at t = 0, t = 48 and t = 72 as estimated using Image J software (NIH).

### Statistical Analysis

Statistical analysis was performed using the Student’s t test.

## Supporting Information

Figure S1 **Anti-Slug antibody validation.** A. CommaDβ cells were untransfected (Ct), or transfected with control (N2), Slug full-length cDNA (SlugS) or Slug antisense full-length cDNA (SlugAS) vectors. Slug protein was found at the expected level (34 kDa) and was dramatically increased in SlugS transfectant. B. CommaDβ cells were also transfected with fusion protein GFP-Slug and Slug-GFP constructs (Savagner et al., J Cell Physiol, 2005, 202∶858), in addition to control N2 vector (CTR EGFPN2). Both native Slug (34 kDa) and fusion protein Slug-GFP or GFP-Slug (60 kDa) were recognized by the anti-Slug antibody. Native Slug was recognized in control N2 cells as a single 34 kDa band, as seen in panel A.
(TIF)

Figure S2 **A.** **Colocalization of Slug and PCNA1 in growing tubule.** Frozen sections of 7 weeks old mouse mammary gland were fixed and processed for immunolocalization to detect Slug and PCNA1. Basal cells expressing Slug were found to also express PCNA1 in a majority of cases (arrowhead). B. Localization of GATA3, Muc1 and Sox 9 were confirmed by co-localization with CK5 or CK8 to be stricly epithelial. GATA3 and Muc1 were found only in luminal cells co-expressing CK8, but not CK5 (arrows). Sox9 was found in both cell types (arrows). Paraffin sections of 10 weeks old mouse mammary gland were used for this immunolocalization after rehydration and antigen retrieval.
(TIF)

Figure S3 **Mammosphere cell analysis.** A. Primary cell mammospheres were fixed after cytocentrifugation and labelled with both anti-cytokeratin antibodies (red) and anti-vimentin antibodies (green). DAPI was also used to locate nuclei. Two examples are shown with a CK- Vimentin+ mammosphere (1) and a CK+Vimentin- mammosphere (2). B. To validate immunolabeling, mammospheres were plated on culture dishes, fixed after 48 h spreading and colabelled with anti-cytokeratin antibodies (green), anti-vimentin antibodies (red) and DAPI. Mammospheres were exclusively epithelial (CK+, vimentin-) or mesenchymal (CK-, vimentin+) in accordance with their clonal origin.
(TIF)

Figure S4 **Slug controls mammary epithelial cell motility.** Cell migration was estimated using a wound healing assay in confluent CommaDβ cells. Cells were transfected as indicated with Slug full length cDNA (in duplicate Slug1 and Slug2) and empty expression vector in duplicate (Ct1 and Ct2), and with anti-Slug siRNA (siSlug1 and siSlug2), and two distinct controls Ct si1 and Ct si2.
(TIF)

Table S1 (PDF)

Table S2 (PDF)
