# Supplementary figures and images for: Slug Controls Stem/Progenitor Cell Growth Dynamics during Mammary Gland Morphogenesis
Source: PLoS One. 2012 Dec 27;7(12):e53498. doi: 10.1371/journal.pone.0053498 (PMC3531397; doi:10.1371/journal.pone.0053498)

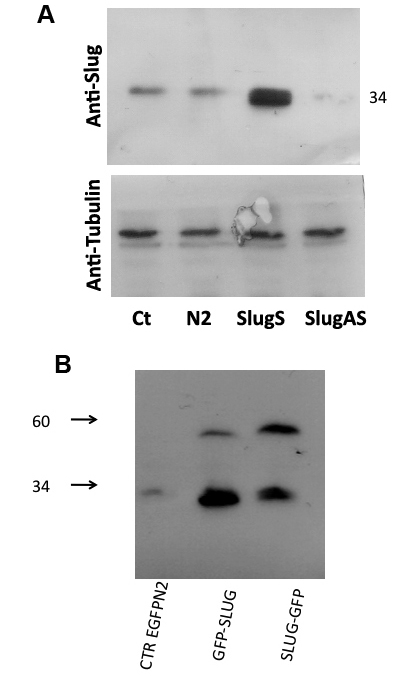

Supplement: Figure S1 — Anti-Slug antibody validation. A. CommaDβ cells were untransfected (Ct), or transfected with control (N2), Slug full-length cDNA (SlugS) or Slug antisense full-length cDNA (SlugAS) vectors. Slug protein was found at the expected level (34 kDa) and was dramatically increased in SlugS transfectant. B. CommaDβ cells were also transfected with fusion protein GFP-Slug and Slug-GFP constructs (Savagner et al., J Cell Physiol, 2005, 202∶858), in addition to control N2 vector (CTR EGFPN2). Both native Slug (34 kDa) and fusion protein Slug-GFP or GFP-Slug (60 kDa) were recognized by the anti-Slug antibody. Native Slug was recognized in control N2 cells as a single 34 kDa band, as seen in panel A. (TIF) [file pone.0053498.s001.tif]

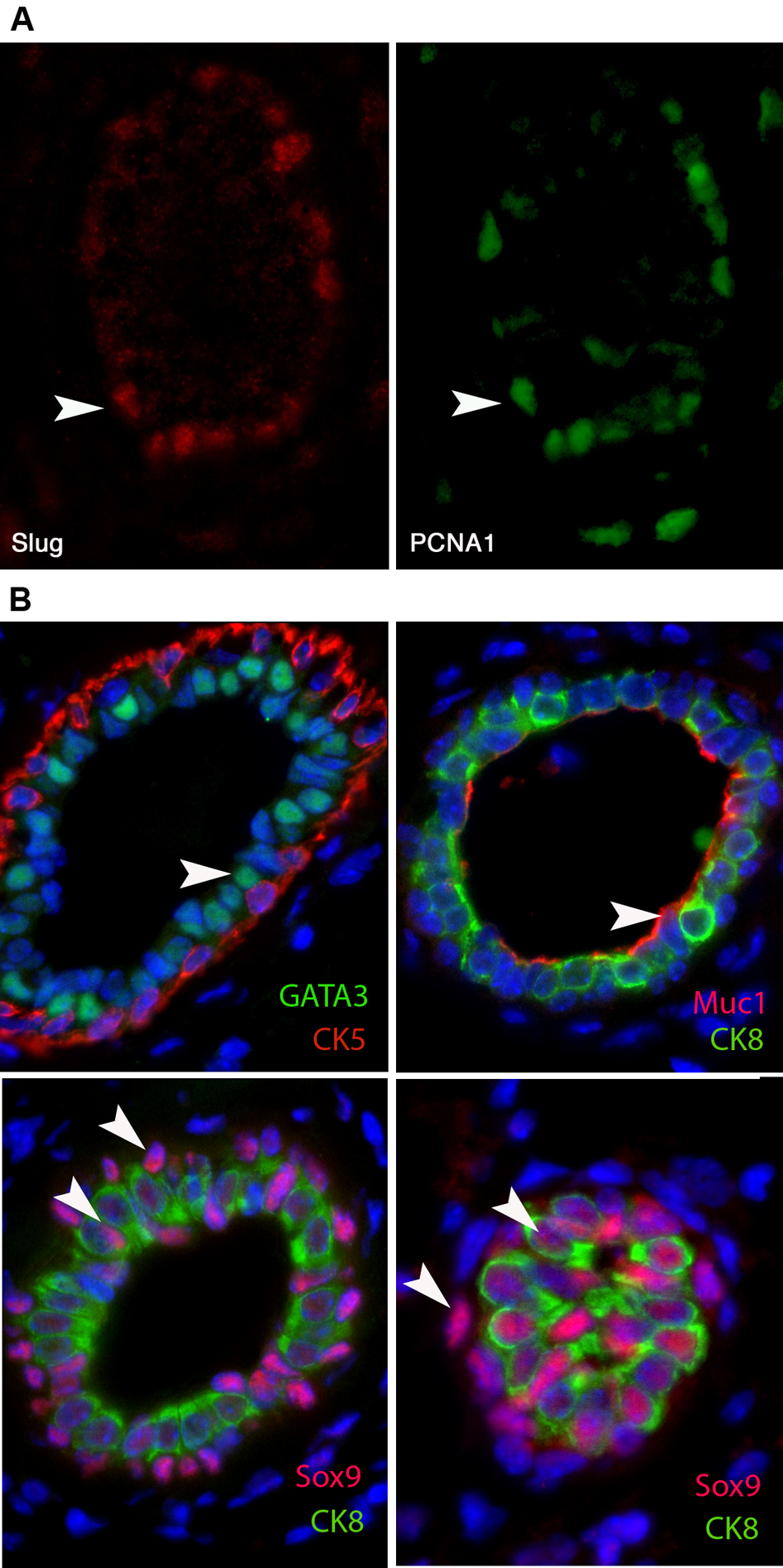

Supplement: Figure S2 — A. Colocalization of Slug and PCNA1 in growing tubule. Frozen sections of 7 weeks old mouse mammary gland were fixed and processed for immunolocalization to detect Slug and PCNA1. Basal cells expressing Slug were found to also express PCNA1 in a majority of cases (arrowhead). B. Localization of GATA3, Muc1 and Sox 9 were confirmed by co-localization with CK5 or CK8 to be stricly epithelial. GATA3 and Muc1 were found only in luminal cells co-expressing CK8, but not CK5 (arrows). Sox9 was found in both cell types (arrows). Paraffin sections of 10 weeks old mouse mammary gland were used for this immunolocalization after rehydration and antigen retrieval. (TIF) [file pone.0053498.s002.tif]

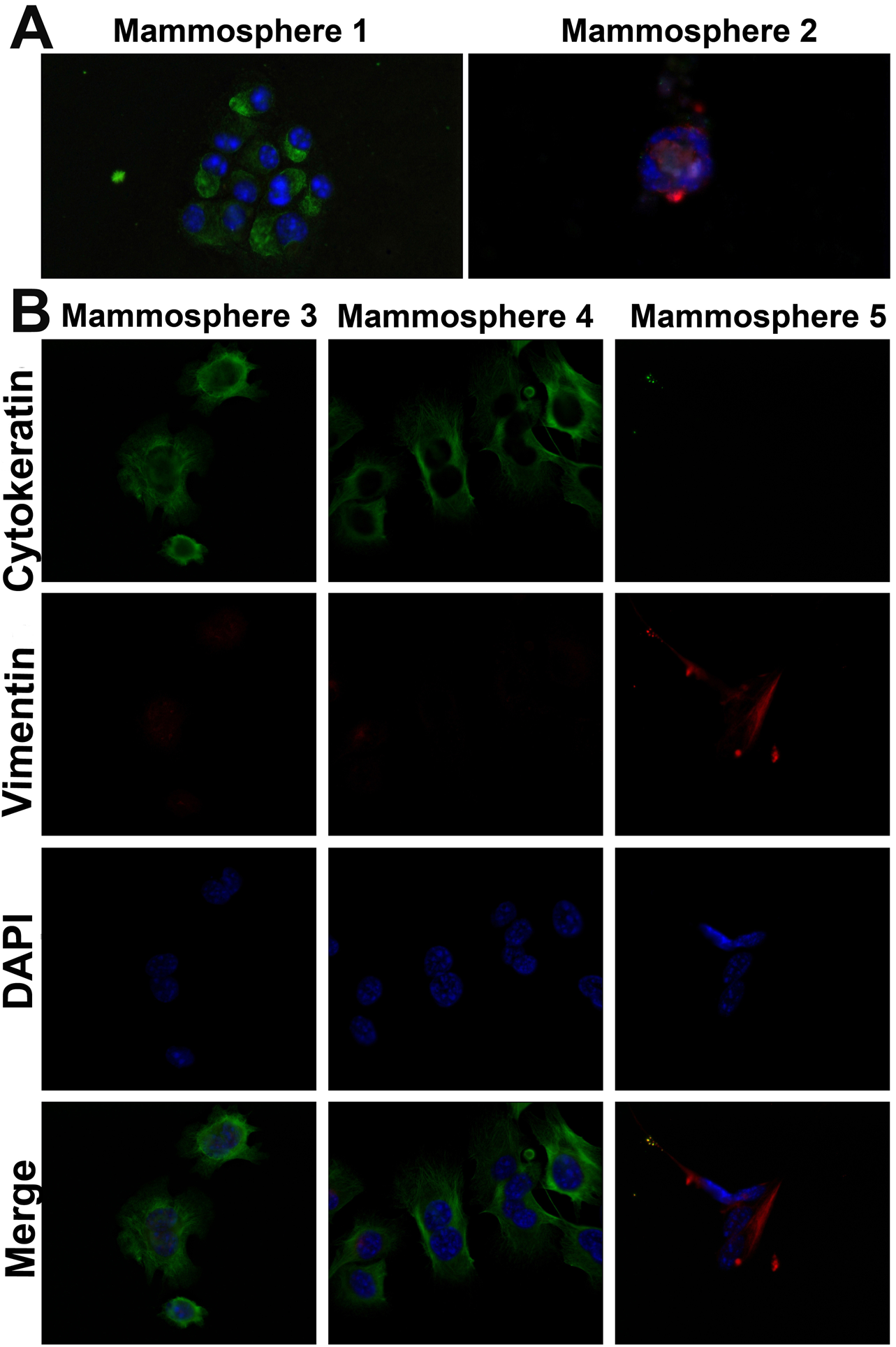

Supplement: Figure S3 — Mammosphere cell analysis. A. Primary cell mammospheres were fixed after cytocentrifugation and labelled with both anti-cytokeratin antibodies (red) and anti-vimentin antibodies (green). DAPI was also used to locate nuclei. Two examples are shown with a CK- Vimentin+ mammosphere (1) and a CK+Vimentin- mammosphere (2). B. To validate immunolabeling, mammospheres were plated on culture dishes, fixed after 48 h spreading and colabelled with anti-cytokeratin antibodies (green), anti-vimentin antibodies (red) and DAPI. Mammospheres were exclusively epithelial (CK+, vimentin-) or mesenchymal (CK-, vimentin+) in accordance with their clonal origin. (TIF) [file pone.0053498.s003.tif]

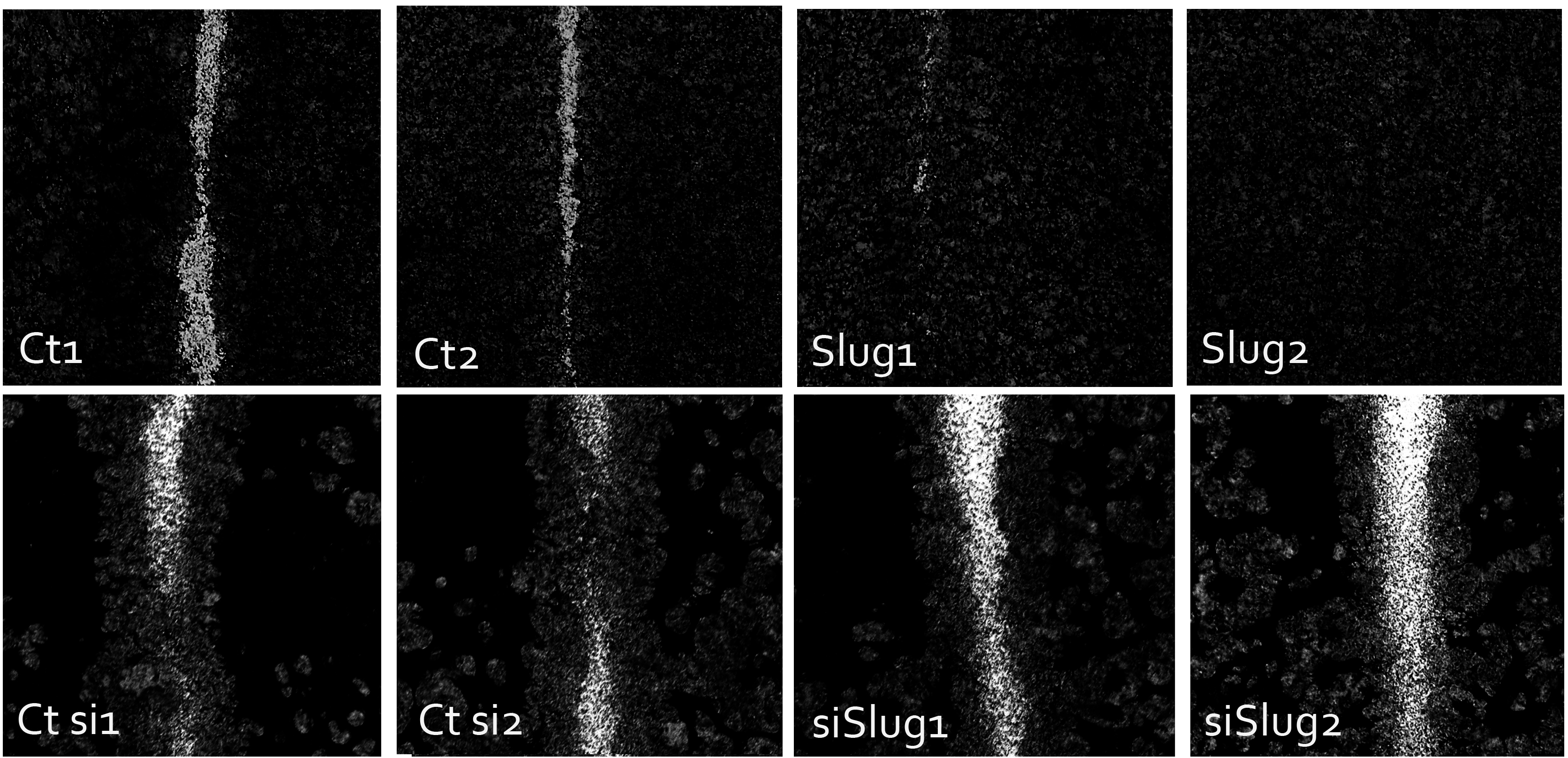

Supplement: Figure S4 — Slug controls mammary epithelial cell motility. Cell migration was estimated using a wound healing assay in confluent CommaDβ cells. Cells were transfected as indicated with Slug full length cDNA (in duplicate Slug1 and Slug2) and empty expression vector in duplicate (Ct1 and Ct2), and with anti-Slug siRNA (siSlug1 and siSlug2), and two distinct controls Ct si1 and Ct si2. (TIF) [file pone.0053498.s004.tif]
