# Supplementary material for: Slug Controls Stem/Progenitor Cell Growth Dynamics during Mammary Gland Morphogenesis
Source: PLoS One. 2012 Dec 27;7(12):e53498. doi: 10.1371/journal.pone.0053498 (PMC3531397; doi:10.1371/journal.pone.0053498)
Supplement: Table S1 — (PDF) [file pone.0053498.s005.pdf]

Nassour-Sup Table1

Antibody origin and methodology

**Antibody**	**Origin**
Alexa Fluor 488	InVitrogen 1/750
Alexa Fluor 555	InVitrogen1/750
Caspase 3	CST 1/100
CD24-PE	Clone M1/69 ; BD Pharmingen
CD31-APC)	Clone MEC13.3; Biolegend
CD45-APC	Clone 30-F11; Biolegend
CD49f-FITC	CloneGoH3 ; BD Pharmingen 1/100
Cytokeratin 5	Covance1/1000
Cytokeratin 8	Covance1/100
GFP	Abcam 1/3000 (Western)
KI67	Abcam Ab15580 1/100
Pcna1	Santa Cruz SC56 1/100
P-cadherin	Zymed 1/100 (1/1000 Western)
Slug	CST 1/100 (1/1000 Western)
SMA	Dako 1/100
Tubulin	Abcam 1/5000 Western
