# Supplementary material for: Slug Controls Stem/Progenitor Cell Growth Dynamics during Mammary Gland Morphogenesis
Source: PLoS One. 2012 Dec 27;7(12):e53498. doi: 10.1371/journal.pone.0053498 (PMC3531397; doi:10.1371/journal.pone.0053498)
Supplement: Table S2 — (PDF) [file pone.0053498.s006.pdf]

Table S2

Gene	NCBI	Forward Primer	Reverse Primer
BMI1	NM_007552.4	TATAACTGATGATGAGATAATAAGC	CTGGAAAGTATTGGGTATGTC
CD24	NM_009846.2	TGCTTCTGGCACTGCTCCTA	CCTCTGGTGGTAGCGTTACTT
CD49f	NM_008397.3	AGAGGGCGAACAGAACAGG	ACGTGCTGCCGTTTCTCAT
CD133	NM_001163577	GCCCAAGCTGGAAGAATATG	CAGCAGAAAGCAGACAATCAA
ECAD	NM_009864.2	AACTGCATGTTTCGAGGTTCT	CATCGCCACAGATGATGGTT
EGFR	NM_007912.4	GCCACGCCAACTGTACCTAT	GCCACACTTCACATCCTTGA
ELF5	NM_010125.3	CCAACGCATCCTTCTGTGAC	AGGCAGGGTAGTAGTCTTCA
ERa	NM_007956.4	TATGCCTCTGGCTACCATTA	ATGGTGCATTGGTTTGTAGC
GATA3	NM_008091.3	TTATCAAGCCCAAGCGAAG	TGGTGGTGGTCTGACAGTTC
ITGB1	NM_010578.2	TGGCAACAATGAAGCTATCG	ATGTCGGGACCAGTAGGACA
ITGB3	NM_016780.2	GGAAGCAGCGCCCAGATCAC	TTGTCCACGAAGGCCCCAAA
KRT5	NM_027011.2	GGTGACCCAGAACCCCAAAA	GTGGGTATCCGGGTAGGGA
KRT8	NM_031170.2	AGTTCGCCTCCTTCATTGAC	GCTGCAACAGGCTCCACT
KRT14	NM_016958.1	CCTCTGGCTCTCAGTCATCC	GAGACCACCTTGCCATCG
KRT18	NM_010664.2	TCTCAACGATGCCCTGGAC	TCTGCCATCCACGATCTTACG
KRT19	NM_008471.2	ACAACAATCTGCCCACCCC	TTTATCACCCCAGTCAGGCC
LGR5	NM_010195.2	GACAATGCTCTCACAGAC	GGAGTGGATTCTATTATTATGG
MUC1	NM_013605.1	CTGTTCACCACCACCATGAC	CTTGGAAGGGCAAGAAAACC
PCAD	NM_001037809	CTACCGAGCGGGCTTCATC	CAGAACTTGGCTCGGCTCC
PR	NM_008829.2	GTCAGGCTGGCATGGTCCTT	AGGGCCTGGCTCTCGTTAGG
SCRT1	NM_130893.3	AGACCTCGACAGCTCCTACG	CCGGATGTGAGGCAGCAGTT
SLUG	NM_011415.2	CCATGCCATACGAGCTGAG	GGCCAGCCCAGAGAACTGA
SMUC	NM_013914.2	GGACAGTACCTCTGCCGTTG	CAGCTGGTTTCCTGAGGTTC
SNAIL	NM_011427.2	ATCTTCCCGGAGCTCACCC	ACCAAGAGAGCCAAGCAGGA
SOX2	NM_011443.3	TCCAAAAACTAATCACAACAATCG	GAAGTGCAATTGGGATGAAAA
SOX9	NM_011448.4	GACAAGCGGAGGCCGAA	CCAGCTTGCACGTCGGTT
SOX10	NM_011437.1	ATGTCAGATGGGAACCCAGA	GTCTTTGGGGTGGTTGGAG
TWIST1	NM_011658.2	GCCCGTGGACAGAGATTC	CTATCAGAATGCAGAGGTGTGG
TWIST2	NM_007855.2	AGTGTTTGGCAGGGGACA	CCCATCCCCTGGGTATCT
VIM	NM_011701.4	CGGCTGCGAGAGAAATTGC	CCACTTTCCGTTCAAGGTCAAG
18s	NR_003278.2	GTTCCGACCATAAACGATGCC	TGGTGGTGCCCTTCCGTCAAT
Pcad promoter (Ebox)		TCGAAGCCAGAGACTGCGAGAG	AACGTAACAAGGTCCCGCCC
Pcad promoter (no Ebox)		AGGCTCGCCCGAAGAGCTGA	GGTTTCGTCTGGCTCCGGGC
Ecad promoter Ebox		GTGGCACGTGCGTTTGCGAG	CCTGCAGGTGGCAGCCAAGG

siSlug1		GGAGCAUACAGCCCUAUUA
siSlug2		GAUGCCCAGUCUAGGAAAU
siCt1		AGGUAGUGUAAUCGCCUUG
siCt2		UAAGGCUAUGAAGAGAUAC
